# Clinical utility of gray scale renal ultrasound in acute kidney injury

**DOI:** 10.1186/1471-2369-14-188

**Published:** 2013-09-08

**Authors:** Amber Podoll, Carl Walther, Kevin Finkel

**Affiliations:** 1 UTHealth Science Center at Houston, Department of Internal Medicine, Division of Renal Diseases & Hypertension, 6431 Fannin MSB 5.134, Houston, TX 77030, USA

**Keywords:** Acute kidney injury, Hydronephrosis, Urinary tract obstruction, Ultrasound

## Abstract

**Background:**

Acute kidney injury occurs commonly in hospitalized patients and is associated with significant morbidity and mortality. Although renal ultrasound is often performed, its clinical utility in determining of the cause of acute kidney injury, particularly the detection of urinary tract obstruction, is not established.

**Methods:**

Retrospective cohort study of all adult inpatients that underwent renal ultrasound for acute kidney injury over a 3-year period at a large university teaching hospital. The frequency of renal ultrasound abnormalities and clinical characteristics that predicted the finding of urinary tract obstruction was determined.

**Results:**

Over the 3-year period, 1471 renal ultrasounds were performed of which 55% (810) were for evaluation of acute kidney injury. Renal ultrasound was normal in 62% (500 of 810) of patients. Hydronephrosis was detected in only 5% (42 of 810) of studies and in only 2.3% (19 of 810) of the cases was obstructive uropathy considered the cause of acute kidney injury. The majority of these patients (14 of 19) had a medical history suggestive of urinary tract obstruction. Less than 1% of patients (5 of 810) had urinary tract obstruction on ultrasound without a suggestive medical history. Most other ultrasound findings were incidental and did not establish an etiology for the acute kidney injury.

**Conclusions:**

Renal ultrasound for evaluation of acute kidney injury is indicated if there is medical history suggestive of urinary tract obstruction. Otherwise, renal ultrasound is unlikely to yield useful results and should be used more selectively based on patients’ medical history.

## Background

Acute kidney injury (AKI) is common among hospitalized patients with a reported prevalence of 2 to 35% [1-3]. The presence of AKI is associated with worse hospital outcome, and even a modest increase in creatinine significantly increases mortality [4].

Evaluation to determine the cause of AKI includes review of the medical history and clinical course, routine blood biochemical measurements, and microscopic examination of the urine. Renal ultrasound (RUS) is often recommended in the evaluation of AKI to exclude the presence of hydronephrosis and urinary tract obstruction even when the pre-test probably for obstructive uropathy is low [5,6]. Others have advocated a more restricted use of RUS given that the majority of cases of AKI in hospitalized patients are due to acute tubular necrosis or prerenal etiologies, and thus in most cases RUS results would not be expected to change management. [7-10] In addition, the finding of hydronephrosis on ultrasound does not prove the presence of urinary tract obstruction since it is also seen in high urinary flow states such as with diuretic use, diabetes insipidus, pregnancy, previous obstruction, and congenital megaureter.

Doppler ultrasonography with determination of resistive indices is an active area of research, with potential diagnostic and clinical implications [11,12], but determination of resistive indices on native renal ultrasound is not yet standard practice.

Reducing the number of unnecessary RUS reduces direct costs. In addition, incidental renal lesions are frequently found, and the benefits of further evaluation and intervention for these incidental findings is unclear and could in some cases lead to harm [8,13,14].

The aim of this study was to assess the clinical utility of RUS in determining the cause of AKI in hospitalized patients. Our goal was to determine the frequency of abnormal findings on RUS, particularly the presence of hydronephrosis, and the clinical characteristics associated with higher likelihood of finding urinary tract obstruction.

## Methods

### Study population

We conducted a retrospective review of all RUS performed from 2004 to 2006 at Memorial Hermann Hospital-Texas Medical Center in Houston. The University of Texas Committee for the Protection of Human Subjects approved this study and granted a waiver of authorization. Patient demographics and pertinent clinical data were obtained from the medical record for the patients who underwent RUS.

All patients who underwent a RUS were identified over the three-year period through an electronic medical record. Patients were excluded from analysis if they were institutionalized, less than 18 years of age, pregnant, recipient of a renal transplant, or clinical data were missing. AKI was defined as a 50% increase in baseline creatinine or a urine output of less than 0.5 ml/kg/hr for more than 12 hours. The etiology of AKI was determined by consensus agreement of the study authors based on review of the medical record. Data collected included demographic and clinical variables. To determine the cause of AKI the presence of contributing factors such as sepsis, hypotension, recent surgery, decompensated heart failure, volume overload, use of intravenous radiocontrast media, and use of nephrotoxic medications were recorded. Diagnoses predisposing to urinary tract obstruction were ascertained for each patient from review of the medical record. Prior abdominal or pelvic malignancy, benign prostatic hypertrophy, nephrolithiasis, ectopic pregnancy, pelvic inflammatory disease, prior pelvic surgery, neurogenic bladder, anatomic genitourinary abnormality, abdominal trauma, and prior renal surgery were considered risk factors for urinary tract obstruction. Obstructive nephropathy was considered the cause of AKI if prompt renal recovery (decrease in serum creatinine within 24 hours) ensued after a urological procedure or bladder catheterization. Renal ultrasound results were obtained from the radiologists’ reports in the electronic medical record.

### Outcomes

A total of 4443 RUS were identified over the three-year period. After applying exclusion criteria, 1471 studies remained, of which 810 studies were performed for the evaluation of AKI, and the remaining 661 were performed for other indications (e.g., evaluation of chronic kidney disease, suspected abscess, hematuria, renal colic, and resistant hypertension).

### Statistical analysis

Comparison of demographic and clinical variables was performed among patients with AKI based on the presence or absence of hydronephrosis on renal ultrasound. Statistical differences between the groups with and without hydronephrosis for the tested dichotomous variables were determined using bivariate logistic regression. A multivariable logistic regression model was created to test the independent associations of clinical and demographic variables with presence of hydronephrosis on RUS. All variables which on bivariate logistic regression analysis had an association with hydronephrosis with P-value less than 0.2 were included in the multivariate model. All statistical analyses were performed using Stata, version 12.1 (StataCorp, http://www.stata.com).

## Results

### Study participants

Characteristics of the 1471 patients who underwent renal ultrasound and did not meet exclusion criteria are listed in Table 1. Patients who underwent RUS for AKI had a mean age of 63.6 years. There were slightly more men in the group (53.2%) and more than one-third were in the intensive care unit (37%). About one-half of the patients who underwent RUS had history of CKD. Only a small proportion of patients with AKI had a history of nephrolithiasis or benign prostatic hypertrophy (1.9%) while a history of abdominal malignancy was found in 8.2%. Neurogenic bladder, retroperitoneal fibrosis, prior ectopic pregnancy, prior pelvic or renal surgery, prior pelvic inflammatory disease, and abdominal trauma were present in a small number of patients (2.8%). The etiologies of AKI determined on clinical grounds are listed in Table 2.

**Table 1:** Characteristics of the patients who underwent renal ultrasound

**Patient characteristics**	**All patients**	**With AKI**
	**(*n *= 1471)**	**(*n *= 810)**
Demographics		
Age (years)	61.1 ± 17.7	63.6 ± 16.4
Male gender	730 (49.6)	431 (53.2)
Clinical data		
ICU admission	374 (25.4)	301 (37.2)
Cr at time of RUS (μM/L)	167 (106–255)	194 (150–290)
Medical history		
CKD	727 (49.4)	444 (54.8)
Nephrolithiasis	35 (2.4)	15 (1.9)
BPH	62 (4.2)	32 (4.0)
Abdominal malignancy	93 (6.3)	68 (8.4)
Other*	55 (3.7)	23 (2.8)

Data are presented as mean ± SD, *n* (%) or median (interquartile range).

Abbreviations: *ICU* intensive care unit, *Cr* creatinine, *RUS* renal ultrasound, *CKD* chronic kidney disease, *BPH* benign prostatic hypertrophy.

*Other includes neurogenic bladder, retroperitoneal fibrosis, known renal or urinary tract anomaly, prior pelvic surgery, prior ectopic pregnancy, prior pelvic inflammatory disease, prior renal surgery, and abdominal trauma.

**Table 2:** Etiology of acute kidney injury

**Etiology**	**n (total 810 patients)**	**% of Total**
Hypotension/prerenal	307	38
Acute tubular necrosis	263	32
Sepsis	50	6
Contrast-induced nephropathy	49	6
Nephrotoxic medication	41	5
Cardiorenal syndrome	21	3
Urinary tract obstruction	19	2
Rhabdomyolysis	14	2
Hepatorenal syndrome	13	2
Glomerulonephritis	13	2
Malignant hypertension	12	1
Thrombotic thrombocytopenic purpura	6	<1
Renal vascular occlusion	2	<1

*ATN* acute tubular necrosis.

### Ultrasound findings

Table 3 summarizes the findings on RUS for patients with AKI. Hydronephrosis was found in 5.2% (42 of 810) patients with an equal number having unilateral and bilateral abnormalities. Increased renal echogenicity was reported in nearly 40% of studies. Simple cysts were the most common anatomic abnormality found on RUS and were present in about 13% (101 of 810) of studies.

**Table 3:** Renal ultrasound results for acute kidney injury

	**(*n *= 810)**
No abnormalities	500 (61.7)
Hydronephrosis	42 (5.2)
Unilateral	21 (2.6)
Bilateral	21 (2.6)
Nephrolithiasis	19 (2.3)
Obstructive	1 (0.1)
Non-obstructive	17 (2.1)
Staghorn	1 (0.1)
Renal parenchymal abnormality (not echogenicity)	49 (6.0)
Increased renal parenchymal echogenicity	320 (39.5)
Anatomic urinary tract abnormality	8 (1.0)
Simple cysts	101 (12.5)
Complex cysts	7 (0.9)
Renal mass	9 (0.9)
Mass of GU tract	1 (0.1)
Other abdominal mass	2 (0.2)
Renal enlargement	6 (0.7)
Renal atrophy or cortical thinning	25 (3.1)
Absence of kidney (whole or partial, congenital or acquired)	13 (1.6)
Pelvic kidney	0 (0)
Horseshoe kidney	1 (0.1)

Data are presented as *n* (%).

Abbreviation: *GU* genitourinary.

### Hydronephrosis

Demographic and clinical characteristics for patients with AKI are presented in Table 4 according to the presence or absence of hydronephrosis. Age 65 years or older, prior diagnosis of abdominal malignancy, and a history of nephrolithiasis were each associated with a higher likelihood of finding hydronephrosis on RUS. The presence of any one of the group of multiple other medical conditions thought to predispose to urinary tract obstruction (neurogenic bladder, retroperitoneal fibrosis, prior ectopic pregnancy, prior pelvic or renal surgery, prior pelvic inflammatory disease, and abdominal trauma) was also associated with a higher likelihood of detecting hydronephrosis on ultrasound.

**Table 4:** Presence of hydronephrosis according to patient characteristics among patients with AKI

**Patient characteristics**	**Hydronephrosis absent (*n *= 768)**	**Hydronephrosis present (*n *= 42)**	**P value†**
Demographics			
Age ≥ 65 years	373 (48.6)	30 (71.4)	0.005
Male sex	412 (53.6)	19 (45.2)	0.29
Medical history			
BPH	29 (3.8)	3 (7.1)	0.284
Abdominal malignancy	58 (7.6)	10 (23.8)	0.001
Nephrolithiasis	12 (1.6)	3 (7.1)	0.018
Other*	16 (2.1)	7 (16.7)	<0.001
CKD	419 (54.6)	25 (59.5)	0.43
Prior normal RUS	168 (21.9)	15 (35.7)	0.04

Data are presented as *n* (%).

Abbreviations: *Cr* creatinine, *RUS* renal ultrasound, *BPH* benign prostatic hypertrophy.

*Other includes neurogenic bladder, retroperitoneal fibrosis, congenital renal or urinary tract anomaly, prior pelvic surgery, prior ectopic pregnancy, prior pelvic inflammatory disease, prior surgery on kidney for reason other than cancer, and abdominal trauma.

†Bivariate logistic regression.

By multivariable logistic regression a history of abdominal cancer was associated with over 3-fold higher odds of finding hydronephrosis in AKI and reached statistical significance. Having a medical condition in the broad group encompassing other factors thought to predispose to urinary tract obstruction (neurogenic bladder, retroperitoneal fibrosis, prior ectopic pregnancy, prior pelvic or renal surgery, prior pelvic inflammatory disease, and abdominal trauma) was associated with 7-fold higher odds of hydronephrosis. Patients aged 65 years or older had over twice the odds of hydronephrosis being found by renal ultrasound in the setting of AKI (Table 5).

**Table 5:** Odds ratios of hydronephrosis on renal ultrasound in AKI based on patient characteristics*

**Patient characteristic**	**Adjusted odds ratio (95% CI)**	**P Value**
Demographics		
Age ≥ 65	2.26 (1.11, 4.62)	0.025
Medical history		
Nephrolithiasis	3.44 (0.76, 15.6)	0.109
Abdominal malignancy	3.25 (1.42, 7.47)	0.005
‡Other	7.73 (2.26, 26.5)	0.001

Abbreviations: *RUS* renal ultrasound, *BPH* benign prostatic hypertrophy, *Cr* creatinine.

*Odds ratios and P values determined by multivariate logistic regression model including variables with bivariate logistic regression P-value < 0.2 (age ≥ 65, nephrolithiasis, abdominal malignancy, and other ‡).

‡Other includes neurogenic bladder, retroperitoneal fibrosis, congenital renal or urinary tract anomaly, prior pelvic surgery, prior ectopic pregnancy, prior pelvic inflammatory disease, prior surgery on kidney for reason other than cancer, and abdominal trauma.

For the patients with AKI who were found to have any hydronephrosis, 54.8% (23 of 42) had known medical conditions predisposing to obstruction. Of the 42 patients with hydronephrosis on RUS, in only 45.2% (19 of 42) was urinary tract obstruction determined to be the cause of AKI. In the remaining patients, hydronephrosis was mild and the cause of AKI was attributed to alternative causes. Of the patients considered to have AKI from urinary tract obstruction, 74% (14 of 19) had at least one of the risk factors associated with obstruction. Thus there were only 0.6% (5 of 810) patients with AKI from urinary tract obstruction without any suggestive medical history, and 4 of these 5 patients were older than 65-years.

### Doppler ultrasonography

Of the 810 renal ultrasounds performed to evaluate for AKI, 336 (41.5%) included Doppler evaluation of renal blood flow. Five of these studies (1.5%) found evidence of renal artery stenosis (RAS), although in none of these cases were those findings thought to contribute to AKI Two studies (0.6%) showed renal artery occlusion, and in both cases this finding had been suspected prior to the study based on the clinical situation. There were no reports of renal vein thrombosis among the Doppler ultrasounds performed for AKI. Resistive indices were reported on only a small number of the studies.

## Discussion

Acute kidney injury is a common occurrence in hospitalized patients and is associated with significantly increased morbidity and mortality [1-3]. Although the most common causes of AKI in this population are sepsis, acute tubular necrosis, and pre-renal states, RUS is often obtained to detect hydronephrosis and possible urinary tract obstruction. However, the indiscriminate use of this procedure increases cost and may expose patients to further unnecessary interventions, and thus restricted use of RUS has been advocated [7-9]. In this retrospective review of RUS use at a large teaching hospital over a 3-year period, we found that over 800 studies were performed for the evaluation of AKI. Hydronephrosis was detected in only slightly more than 5% of patients, and in less than half of them (2.3%) was the cause of AKI attributable to urinary tract obstruction. More often the hydronephrosis was mild and considered an incidental finding. This observation is consistent with a previous study that showed that 11% of patients with AKI were found to have hydronephrosis on RUS unrelated to their renal failure [15].

Doppler evaluation of renal blood flow was performed in almost half of the renal ultrasounds, and resulted in only 7 additional clinically relevant findings. It is notable, however, that resistive indices are not routinely reported on native kidney renal ultrasounds at our institution, so we are unable to comment on the utility of this metric which may provide prognostic and diagnostic information in AKI [11,12].

We found that several characteristics were independently associated with the finding of hydronephrosis by RUS in AKI. Age 65 or greater, prior abdominal malignancy, or any one of a grouping of several factors that could affect the urinary tract (neurogenic bladder, retroperitoneal fibrosis, prior ectopic pregnancy, prior pelvic or renal surgery, prior pelvic inflammatory disease, and abdominal trauma) were all associated with an statistically significant increased likelihood of detecting hydronephrosis. This is consistent with the previously reported results of Licurse et al. showing that history of abdominal anatomic abnormality, BPH, or neoplasia conferred a significantly higher risk of finding hydronephrosis on renal ultrasound [8].

It has been suggested that including RUS in evaluation of most cases of AKI is important to avoid missing cases of significant urinary tract obstruction [16]. However the results of our study suggest that a more targeted approach may be practical. Of the 19 cases of urinary tract obstruction resulting in AKI, only 5 cases had no known medical condition predisposing to urinary tract obstruction, and four of these 5 patients were over age 65. Similar findings, among patients with AKI in an intensive care unit, were reported by Keyserling et al., who found that of 100 renal ultrasounds performed for AKI in patients without clinical findings suggestive of obstruction, only one case of hydronephrosis was found [17]. In our sample, if RUS had been ordered only in patients with a predisposing medical conditions or age greater than 65, 44% (360 of 810) fewer ultrasounds would have been performed while missing only a single case of urinary tract obstruction.

The intent of the study was to refine the criteria for obtaining a RUS in the evaluation of AKI. It does not suggest that findings of non-obstructive hydronephrosis, kidney size, and cortical echogenicity and thickness are not useful in determining the presence of chronic kidney disease in the setting of renal insufficiency. Rather, obtaining these results early in the course of suspected AKI is unlikely to change initial management, and delaying RUS until the clinical course suggests underlying chronic renal dysfunction might result in fewer unnecessary ultrasounds*.* In addition, we are unable to comment on the utility of renal Dopplers in the evaluation of AKI because the test was obtained in less than 50% of subjects and there were no specific indications for its performance. Further, resistive indices were rarely reported. Whether there is a specific role for Doppler examination of renal blood flow in evaluating AKI remains an area of active investigation.

The strengths of this study include the large number of patients. The study was not intended to determine the overall incidence of urinary tract obstruction as a cause of AKI since there are other imaging techniques to make the diagnosis. We were interested solely in the utility of RUS in the evaluation of AKI as used in current practice. The main weaknesses of our study are the retrospective nature and the single institutional setting which may lead to selection bias. Further bias may have been introduced by excluding cases of AKI due to obstruction which were diagnosed by other radiologic methods. Finally, since our study indicates that medical history may reduce the rate of unnecessary RUS, our results may not apply when medical history is unavailable.

## Conclusions

Renal ultrasound is frequently used in the evaluation of AKI. Our study showed that over a three-year period at a large teaching hospital, RUS performed in evaluation of AKI revealed findings which changed management almost exclusively in patients with clinical factors predisposing to urinary tract obstruction. Limiting use of RUS to these patients may reduce the number of unnecessary tests while still identifying nearly all cases of clinically important urinary tract obstruction. By applying certain clinical characteristics RUS utilization may be reduced in the evaluation of AKI without significant risk to patients.

## Abbreviations

AKI: Acute kidney injury; ATN: Acute tubular necrosis; BPH: Benign prostatic hypertrophy; CKD: Chronic kidney disease; RUS: Renal ultrasonography.

## Competing interests

The authors have no competing interests to declare.

## Authors’ contributions

All 3 authors contributed equally to the study design, analysis of the data, and the preparation of the manuscript. No financial support was provided for this study. The authors have no financial disclosures to make. All authors read and approved the final manuscript.

## Pre-publication history

The pre-publication history for this paper can be accessed here:

http://www.biomedcentral.com/1471-2369/14/188/prepub
